# Effects of Physical Rehabilitation and Nutritional Intake Management on Improvement in Tongue Strength in Sarcopenic Patients

**DOI:** 10.3390/nu12103104

**Published:** 2020-10-12

**Authors:** Ayano Nagano, Keisuke Maeda, Masaki Koike, Kenta Murotani, Junko Ueshima, Akio Shimizu, Tatsuro Inoue, Keisuke Sato, Masaki Suenaga, Yuria Ishida, Naoharu Mori

**Affiliations:** 1 Department of Nursing, Nishinomiya Kyoritsu Neurosurgical Hospital, 11-1 Imazuyamanaka-cho, Nishinomiya, Hyogo 663-8211, Japan; aya.k.nagano@gmail.com; 2 Department of Geriatric Medicine, National Center for Geriatrics and Gerontology, 7-430 Morioka, Obu, Aichi 474-8511, Japan; 3 Department of Palliative and Supportive Medicine, Graduate School of Medicine, Aichi Medical University, 1-1 Yazakokarimata, Nagakute, Aichi 480-1195, Japan; nmori@aichi-med-u.ac.jp; 4 Division of Rehabilitation, Chuzan Hospital, 6-2-1 Matsumoto, Okinawa 904-2151, Japan; koikemasa4724@gmail.com; 5 Biostatistics Center, Kurume University, 67 Asahimachi, Kurume 830-0011, Japan; kmurotani@med.kurume-u.ac.jp; 6 Department of Clinical Nutrition and Food Service, NTT Medical Center Tokyo, 5-9-22 Higashi-Gotanda, Shinagawa-ku, Tokyo 141-8625, Japan; j.ueshima@gmail.com; 7 Department of Nutrition, Hamamatsu City Rehabilitation Hospital, 1-6-1 Wago-kita, Naka-ku, Hamamatsu, Shizuoka 433-8127, Japan; a.shimizu.diet@gmail.com; 8 Department of Physical Therapy, Niigata University of Health and Welfare, 1398 Shimami-cho, Kita-ku, Niigata 950-3198, Japan; tatsuro-inoue@nuhw.ac.jp; 9 Okinawa Chuzan Hospital Clinical Research Center, Chuzan Hospital, 6-2-1 Matsumoto, Okinawa 904-2151, Japan; keisuke.sato0815@gmail.com; 10 Department of Rehabilitation Medicine, Chuzan Hospital, 6-2-1 Matsumoto, Okinawa 904-2151, Japan; suemasa0916@gmail.com; 11 Department of Nutrition, Aichi Medical University, 1-1 Yazakokarimata, Nagakute, Aichi 480-1195, Japan; okuda.yuria.785@mail.aichi-med-u.ac.jp

**Keywords:** tongue pressure, sarcopenia, sarcopenic dysphagia, nutritional care

## Abstract

The study aimed to investigate the impact of physical intervention and the amount of nutritional intake on the increase in tongue strength and swallowing function in older adults with sarcopenia. From November 2018 and May 2019, older patients with sarcopenia who were admitted for rehabilitation were analyzed. The intervention employed in the study was the usual physical and occupational therapy for two months. Tongue strength was measured before and after two months of treatment. Data on tongue strength, the amount of energy and protein intake, intervention time, and swallowing function were examined. A total of 95 sarcopenic older patients were included (mean age 83.4 ± 6.5 years). The mean tongue strength after the intervention was significantly increased from 25.4 ± 8.9 kPa to 30.5 ± 7.6 kPa as a result of the treatment (*p* < 0.001). After adjusting the confounding factors in the multivariable models, an energy intake of ≥30 kcal/kg/day and a protein intake of ≥1.2 g/kg/day based on the ideal body weight had a significant impact on the increase in tongue strength after the treatment (*p* = 0.011 and *p* = 0.020, respectively). Swallowing function assessed using the Mann Assessment of Swallowing Ability was significantly increased after the treatment (mean difference between pairs: 1.12 [0.53–1.70]; *p* < 0.001). Physical intervention and strict nutritional management for older inpatients with sarcopenia could be effective to improve tongue strength and swallowing function.

## 1. Introduction

Sarcopenia is a condition characterized by a decline of muscle mass and muscle function and causes various health problems in older adults [1]. Sarcopenia is related to an increased incidence of falls, fractures, and mortality [2,3,4,5]. Given the various negative impacts of sarcopenia on body composition and physical health, the quality of life of sarcopenia patients will also likely be compromised [2]. Moreover, sarcopenia occurs in the swallowing muscles and leads to swallowing dysfunction [6,7,8,9,10,11,12], which is referred to as sarcopenic dysphagia. Sarcopenic dysphagia is characterized by a sarcopenia-induced swallowing disorder and the loss of swallowing muscle mass and function [6].

Sarcopenic dysphagia has received increased attention in recent studies [10,11,12,13,14]. The prevalence of sarcopenic dysphagia is reported to be 13% in older women after hip fracture surgery [12], 33% in hospitalized older patients with sarcopenia [10], and 32% in inpatients who have undergone dysphagia rehabilitation [11]. The risk factors of sarcopenic dysphagia include hospitalization, low physical function, malnutrition, and severe sarcopenia [10,12,13,14]. Decreased hand-grip strength, decreased skeletal muscle mass, and sarcopenia are associated with decreased tongue strength [15,16,17,18,19]. In addition, decreased tongue strength is associated with dysphagia [18,20,21], and it is demonstrated in older people with sarcopenic dysphagia [17]. An algorithm to diagnose sarcopenic dysphagia has been proposed, in which muscle strength is an indicator of swallowing muscle strength [6,22].

There has been a lack of evidence for sarcopenic dysphagia interventions [6,23]. Training methods that directly focus on the swallowing muscles such as head lifting exercises or tongue strength training are reported to be effective to improve tongue strength and swallowing function [24,25,26,27,28]. Wakabayashi et al. [29] have also reported that resistance training of the swallowing muscles tends to improve dysphagia in subjects with sarcopenic dysphagia compared with that in subjects with dysphagia that is caused by other factors such as stroke. Moreover, several case reports have indicated that intervention for whole-body sarcopenia can be effective for sarcopenic dysphagia, in addition to dysphagia training [30,31,32,33]. In these case reports, strict nutritional support was provided along with systemic rehabilitation [30,31,32].

Physical and nutritional interventions may improve tongue strength, because sarcopenic dysphagia occurs along with whole-body sarcopenia, and sarcopenia-related factors are associated with the development of sarcopenic dysphagia [6,12,13]. Swallowing is a complex behavior that involves the activities of many nerves and muscles. The swallowing process is commonly divided into oral, pharyngeal, and esophageal stages. The tongue is a major muscle that participates in the transition from the oral phase to the pharyngeal phase of swallowing. Tongue strength is one of the important factors in swallowing. Thus, improvements in tongue strength could be associated with improvements in swallowing function [25,28]. However, the effectiveness of physical and nutritional interventions for sarcopenic dysphagia has not been clarified. This study aimed to investigate the impact of physical intervention and the amount of nutritional intake on increasing tongue strength for older adults with sarcopenia who were not receiving swallowing rehabilitation. This study implies that standard physical intervention and strict nutritional management can be effective in improving tongue strength and swallowing function in older patients with sarcopenia.

## 2. Materials and Methods

### 2.1. Ethics

The single-arm intervention study was approved to perform its protocol by the local institutional review board of Chuzan Hospital and was conducted according to the Declaration of Helsinki. Informed consent from all the participants was obtained before the intervention (ID: 18-183). The study was registered in the UMIN clinical trials registry before initiation of the study (UMIN000034900).

### 2.2. Study Design and Participants

This study was conducted in a rehabilitation hospital with six convalescent rehabilitation wards. The hospital provides in-hospital rehabilitative training and care based on the Japanese health insurance system [34]. The hospital has 216 beds for the convalescent rehabilitation wards. Between November 2018 and May 2019, newly admitted patients who underwent rehabilitation for orthopedic diseases/conditions, such as hip fracture and vertebral compression fracture, were examined for eligibility, and if eligible, patients were included in the study. The inclusion criterion was patients with sarcopenia aged ≥65 years. Patients who were undergoing prescribed dysphagia rehabilitation with speech therapists, those with contraindications for bioimpedance analysis (BIA) measurement such as a pace-maker, those with an error that occurred during BIA measurement, and those with a disability that affected compliance with the measurement of tongue strength and the assessment of swallowing function were excluded. Further, patients with an unexpected discharge due to being transferred to other hospitals to treat complicated acute diseases were excluded.

### 2.3. Physical Intervention

The intervention employed in the study was the usual physical and occupational therapy for hospitalized patients based on the Japanese health insurance system [34]. Participants received no other experimental treatment or training. Generally, the types and amounts of the therapy were determined by the attending physical therapists and occupational therapists according to the results of the physical assessment that was evaluated by the therapists and nurses, the nutritional assessment that was evaluated by a registered dietician deployed to the ward, and other information that included environmental and individual factors. The physicians provided supervision and managed the care and provided preventive and therapeutic treatment for comorbid diseases. To provide convalescent rehabilitation for hospitalized patients, the health insurance system requires one or more meetings per month with the multidisciplinary staff such as physicians, nurses, rehabilitation therapists, and registered dietitians. The multidisciplinary assessment and meetings may contribute to the individualized and condition-adjusted care [34]. Dysphagia rehabilitation for the swallowing muscles was not provided because no speech therapy intervention was prescribed for the analyzed patients included in the study. During the study, the duration of the intervention in minutes was separately recorded by the physiotherapists and occupational therapists.

### 2.4. Tongue Pressure Measurement

The tongue strength was measured as the maximum voluntary tongue pressure against the palate by speech therapists using a device that consisted of a disposable oral balloon probe (JMS tongue pressure measuring instrument, JMS, Hiroshima, Japan) as previously described [17]. The participants were placed in a relaxed sitting position and then were asked to place the balloon in their mouth and to hold the plastic pipe at the midpoint of their central incisors with their lips closed. For the measurement, the participants were instructed to compress a small balloon, attached to the tip of the probe, between the tongue and the anterior part of the hard palate for 5 s with maximum voluntary effort. This instrument is widely used to evaluate tongue weakness within the diagnostic criteria for oral frailty and is supported by the Japanese health insurance system [34]. The pressures were measured twice, and the higher value was recorded. The tongue strength examination was performed before and after two months of intervention. If a participant was discharged from the hospital within two months, the tongue strength was measured at discharge. Tongue pressure <20 kPa is considered to be a decreased tongue strength for sarcopenic older adults [6].

### 2.5. Dysphagia and Physical Function Assessment Method

Physical and swallowing conditions were evaluated using the Functional Independence Measure (FIM) and the Mann Assessment of Swallowing Ability (MASA) instruments, respectively. The FIM reflects activities of daily living and consists of 13 motor function and five cognitive function items [35]. Ottenbacher et al. showed that the FIM demonstrated acceptable reliability across a wide variety of settings, raters, and patients (a median inter-rater reliability for the total FIM of 0.95 and median test–retest and equivalence reliability values of 0.95 and 0.92, respectively) [35]. For each item, the independence level is evaluated in seven stages from total assistance (1 point) to complete independence (7 points), and the total score ranges from 18 to 126 points. The MASA is a comprehensive and simple evaluation tool for swallowing ability, which consists of 24 items related to swallowing function and symptoms, and the scored sum ranges from 0 to 200 points [36]. A score of 178 points or less indicates a high risk for aspiration; the sensitivity and specificity of the tool were reported to be 73% and 89%, respectively, with good inter- and intra-rater reliability [36].

### 2.6. Nutritional Intake Assessment Method

For nutritional variables, body mass index, defined as the calculated actual body weight [kg] divided by the height [m] squared, the Mini Nutritional Assessment Short Form (MNA-SF), the skeletal muscle mass index (SMI), and hand-grip strength were obtained. The MNA-SF is a nutritional risk screening tool for older adults, which ranges from 0 to 14 points, and a score of 11 or less indicates existing nutritional risk [37]. The SMI was calculated from the appendicular skeletal muscle mass based on the BIA analysis divided by the height [m] squared. Hand-grip strength was measured in a sitting position with a 90-degree bend of the elbow using a Jamar type measurement instrument (MG-4800, MORITOH co., Aichi, Japan). The highest value of the right and left hand was recorded in kg with one decimal place. Sarcopenia diagnosis was performed according to the latest criteria from the Asian Working Group for Sarcopenia, while the study participants were enrolled, which required both a low SMI (<7.0 kg/m^2^ for men and <5.7 kg/m^2^ for women) and a decreased hand-grip strength (<26 kg for men and <18 kg for women) [38].

### 2.7. Amount of Nutrition

Ward-deployed dietitians calculated daily energy and protein intake values based on the nurses’ and dietitians’ records. They recorded a visual estimate of the percentage of each meal that the participants ingested. Although the amount of energy and protein intake values were not directly measured, the amount of nutrition in the provided meals was able to be obtained because of the standard foodservice practice of the hospital to plan a diet menu based on the Standard Tables of Food Composition in Japan—2015, 7th Revised Edition [39] by employing commercial meal planning software.

### 2.8. Outcome Measures

The primary outcome was a change in the tongue strength before and after the intervention. Changes in the MASA and FIM were also considered secondary outcomes. The MASA was assessed by speech therapists and the FIM was assessed by the nurses. The MASA and the FIM were scored by the attending speech therapist or the attending nurse at baseline and after two months of intervention or discharge within two months.

### 2.9. Sample Size Calculation

We calculated the sample size to detect the tongue strength changes based on the following assumption. There was no interventional study related to this study; however, an effect size of 0.6 was shown in a study that reported tongue strength training [40]. Since the intervention used in this study might be less effective than direct tongue strength training, we assumed that the effect size was expected to be at least half that of direct training. Therefore, we set the effect size at 0.3 for the sample size calculation. To obtain an alpha error of 0.05 and a power of 0.80, 90 or more participants were required. Finally, we examined 150 older adult patients for eligibility regarding non-sarcopenia, failure to conduct the tongue strength measurement, and the occurrence of other incidences that belonged to the exclusion criteria.

### 2.10. Statistical Analysis

Quantitative variables were presented as mean ± standard deviation, and differences were analyzed by a paired *t*-test for parametric variables in the histograms. Non-parametric variables in the histograms were expressed as a median [interquartile range]. Categorical data were expressed as a number (percentage). Linear regression analysis was used to estimate the impact of each variable on tongue strength after the intervention adjusted by tongue strength before the intervention. Considering that an energy intake of ≥30 kcal/kg/day and a protein intake of ≥1.2 g/kg/day based on the ideal body weight was suitable to recover from sarcopenic dysphagia [6,30], whether the participants consumed the nutritional intake was investigated, and its impact on the tongue strength change was examined in multivariate analyses. Multivariable linear regression analyses were performed to determine whether nutritional load and physiotherapy contributed to the change in tongue strength. Confounding factors in the multivariable models were chosen from the baseline variables if a *p* value < 0.2 in each linear regression model was obtained. Values of *p* < 0.05 were considered statistically significant. The analysis was performed using SPSS 23.0 software (IBM Japan, Tokyo, Japan).

## 3. Results

A total of 150 sarcopenic older adults were examined to determine their eligibility to participate in the study. After applying the exclusion criteria, 55 patients were excluded; three were prescribed dysphagia rehabilitation, three had contraindications for BIA, 44 were unable to comply with the instruction, and five were unexpectedly discharged. Finally, 95 patients were included in the analysis. The baseline characteristics of the patients are depicted in Table 1. Decreased tongue strength was detected in 30 patients (31.5%). The mean tongue strength, MASA, and FIM of all participants at baseline were 25.4 ± 8.9 kPa, 191.8 ± 5.7 points, and 65.9 ± 14.5 points, respectively. The mean hand grip strength and SMI at baseline were 13.7 ± 4.7 kg (17.9 ± 6.5 kg for men and 12.7 ± 3.4 kg for women) and 4.9 ± 0.8 kg/m^2^ (5.8 ± 0.7 kg/m^2^ for men and 4.6 ± 0.7 kg/m^2^ for women), respectively. The mean length of day from onset was 17 days.

Table 2 describes the amount of nutritional intake and the intensity of the intervention. There was a narrow distribution of the intervention time per day recorded by the physical and occupational therapists (mean: 129.6 ± 18.1 min). Most patients consumed nutrition properly and received the intervention for two months (median length of days: 60 [53–60]).

**Figure 1:**
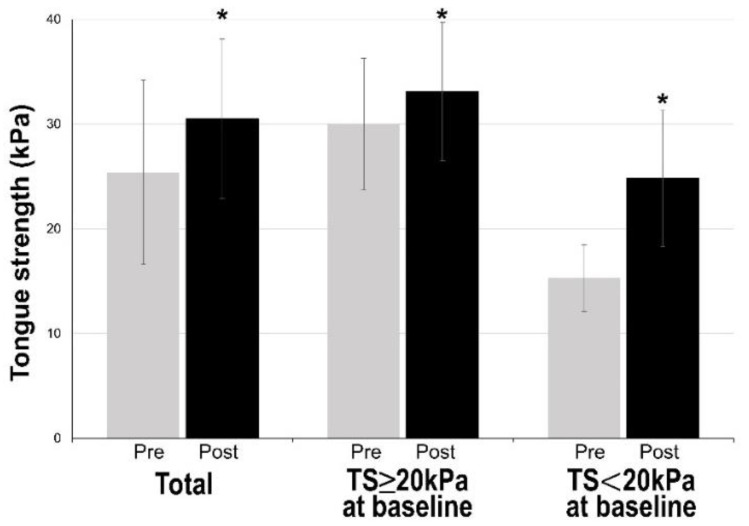
Changes in tongue strength before and after the intervention. Tongue strength after the intervention was 30.47 ± 7.64 kPa, which was significantly increased compared with that at baseline (*p* < 0.001). In the patient groups classified according to tongue strength at baseline ≥20 kPa and <20 kPa, tongue strength after the intervention was 33.10 ± 6.64 kPa and 24.79 ± 6.64 kPa, respectively, and it was significantly increased compared with tongue strength at baseline (*p* < 0.001) in both groups. Abbreviations: TS, tongue strength; * *p* < 0.001 by paired *t*-test.

Changes in tongue strength are shown in Figure 1. Tongue strength after the intervention was 30.47 ± 7.64 kPa, which was significantly increased compared with that at baseline (*p* < 0.001). In the patient groups classified by tongue strength at baseline ≥20 kPa and <20 kPa, tongue strength after the intervention was 33.10 ± 6.64 kPa and 24.79 ± 6.64 kPa, respectively, and it was significantly increased compared with tongue strength at baseline (*p* < 0.001) in both groups.

Changes in other outcomes are shown in Table 3. The MASA and FIM total scores were significantly increased after the intervention (mean difference between pairs: 1.12 [0.53–1.70]; *p* < 0.001, and 30.9 [28.1–33.7] *p* < 0.001, respectively). In patients with decreased tongue strength (<20 kPa), the MASA and FIM total scores were also significantly increased after the intervention (mean difference between pairs: 3.00 [1.71–4.23]; *p* < 0.001, and 30.9 [25.9–35.9] *p* < 0.001, respectively).

Male sex (*p* = 0.123), hand-grip strength (*p* = 0.003), SMI (*p* = 0.043), and MASA at admission (*p* = 0.028) were selected as confounding factors in the multivariable models (Table 4). After adjustment by the confounding factors, high energy intake and high protein intake demonstrated a significant impact on the tongue strength increase after the intervention (*p* = 0.011 and *p* = 0.020, respectively). Physical therapy intervention time per day had no impact on the tongue strength change (*p* = 0.575).

## 4. Discussion

This study aimed to investigate the impact of physical intervention and the amount of nutritional intake, without swallowing rehabilitation, on the increase in tongue strength. This study revealed two notable findings. First, tongue strength in rehabilitation patients with orthopedic conditions increased under the usual physio- and occupational therapies. Second, a high amount of energy and protein intake were associated with an increase in tongue strength.

Tongue strength in rehabilitation patients increased under the usual physical intervention, without swallowing rehabilitation. Physical intervention is effective to improve muscle strength and increase the muscle mass of the body [41,42,43,44,45]. Furthermore, hand-grip strength and skeletal muscle mass are associated with tongue strength [15,16,17,18,19]. Voit et al. [46] have reported that the prescription of leg-specific exercise improves whole-body strength. Regarding this connection, physical intervention may affect tissues throughout the body, including swallowing-related muscle groups [23,47]. The direct relationship between tongue strength and whole-body muscle mass or strength is unclear; however, physical intervention improved tongue strength in the current study. Tongue strength is one of the indicators of swallowing function. Oral and pharyngeal function, as well as posture and durability, are associated with swallowing [48]. Thus, the improvement of whole-body integrity by the physical intervention was thought to affect the tongue strength increase and improvement of the swallowing function.

High energy and high protein intake were associated with increased tongue strength. Nutritional intervention is effective to treat whole-body sarcopenia [45]. Strict nutritional intervention with 32–35 kcal/day based on the ideal body weight was conducted in case reports on sarcopenic dysphagia [30,31,32]. High energy intake of over 30 kcal/day based on the ideal body weight was independently associated with an increase in tongue strength in the current study. On the other hand, muscle protein synthesis requires a sufficient amount of protein intake due to anabolic resistance in older people [49]. The expert group of the European Society for Clinical Nutrition and Metabolism recommends a protein intake of 1.2–1.5 g/kg/day. In addition, a society of Sarcopenia, Cachexia, and Wasting Disorders’ Position Paper recommends a protein intake of over 1.5 g/kg/day combined with exercise [45]. The current study supports these recommendations. This study showed that a protein intake of ≥1.2 g/kg/day was independently associated with an increase in tongue strength. Thus, a high amount of energy and protein intake could be effective to improve the swallowing function of older people with sarcopenic dysphagia.

Physical intervention that is combined with strict nutritional management along with high energy and protein intake may be essential for people with sarcopenic dysphagia. Malnutrition and low physical activity are the main factors that cause sarcopenia. On the other hand, sarcopenia also leads to malnutrition and low physical activity that can result in a vicious cycle. Therefore, a combined physical and nutritional intervention can be effective, and it is recommended for the treatment of sarcopenia [50] Sarcopenic dysphasia is characterized by a worsening of swallowing function as a result of muscle mass loss and muscle weakness; consequently, the same interventions as those for whole-body sarcopenia can be effective [6]. Not only for sarcopenic dysphagia, physical intervention [51] and nutritional care [52] can be effective for stroke or head and neck cancer patients. Yoshimura et al. [51] showed the effects of a chair stand exercise on the swallowing function in 148 stroke patients with dysphagia admitted to a rehabilitation hospital (mean age 72.7 years; 48.6% men). In multivariate analyses, the frequency of chair stand exercise was independently associated with the swallowing function at discharge (β = 0.231, *p* = 0.015) and the presence of dysphagia at discharge (odds ratio: 0.982, *p* = 0.035). In the study above, the sole impact of physical intervention on the improvement of swallowing function remained unclear, as the patients were also prescribed dysphagia rehabilitation. Daily intake of 1.2 g/kg protein and at least 30 kcal/kg energy have been recommended for patients with head and neck cancer [52]. However, these recommendations were estimated for non-obese ambulatory patients using their actual body weight, not from an intervention study. In the current study, we revealed that the daily intake of 1.2 g/kg protein and 30 kcal/kg energy are necessary to improve tongue strength for the older patients with sarcopenic dysphagia. Recently, photobiomodulation therapy is drawing attention to be effective for the adverse effects of treatment for head and neck cancer, including dysphagia [53]. Future investigations should be conducted to better define optimal photobiomodulation parameters for dysphagia in head and neck cancer treatment. The current study implies that standard physical intervention and strict nutritional management can be effective to improve tongue strength and swallowing function for older patients with sarcopenia.

The current study has several limitations. First, this was a single-armed intervention study. However, it was ethically impossible to include a control group of patients with dysphagia who did not receive swallowing training. Thus, to clarify the efficacy of physical intervention, we recruited patients who were undergoing rehabilitation for orthopedic diseases and who had conditions that did not need swallowing rehabilitation. Careful consideration is necessary in that these results were not obtained due to the natural and physiological improvement in the patients. Second, this study did not include patients with sarcopenic dysphagia. Further studies that target sarcopenic dysphagia should be conducted. Moreover, it is not clear if an energy intake of ≥30 kcal/kg/day and a protein intake of ≥1.2 g/kg/day based on the ideal body weight are sufficient to treat sarcopenic dysphagia. Third, in the study, swallowing function was evaluated by a screening assessment tool, not by an instrumental assessment such as videofluorography. Further studies with an instrumental swallowing assessment are needed to precisely evaluate the change in swallowing function regarding the impact of intervention. Finally, this was a single-center study, which could limit the generalization of the results. Further studies regarding the impact of physical intervention and nutritional management on sarcopenic dysphagia should be conducted including larger cohorts at multiple centers and possibly in different Asian countries. A heterogeneous population should also be included in future studies.

## 5. Conclusions

The current study provided evidence that physical intervention and strict nutritional management for older inpatients with sarcopenia could be effective to improve tongue strength and swallowing function. Physical intervention with strict nutritional management could be added to swallowing exercise to treat sarcopenic dysphagia.

## Figures and Tables

**Table 1:** Baseline characteristics at admission to the rehabilitation hospital.

Variable	All (*n* = 95)	Female (*n* = 76)	Male (*n* = 19)
Age, years	83.4 ± 6.5	84.3 ± 5.0	79.3 ± 7.2
BMI, kg/m^2^	21.6 ± 2.9	21.9 ± 3.0	20.7 ± 2.7
MNA-SF, score	7 [6–9]	7 [6–9]	7 [5–8]
Hand-grip strength, kg	13.7 ± 4.7	12.7 ± 3.4	17.9 ± 6.5
SMI, kg/m^2^	4.9 ± 0.8	4.6 ± 0.7	5.8 ± 0.7
MASA, score	191.8 ± 5.7	191.8 ± 6.2	191.8 ± 3.4
FIM total, score	65.9 ± 14.5	64.9 ± 13.9	70.0 ± 16.3
Motor	42.0 ± 11.7	41.4 ± 11.3	44.2 ± 13.0
Cognitive	23.9 ± 5.2	23.5 ± 5.2	25.6 ± 5.3
Tongue strength, kPa	25.4 ± 8.9	24.9 ± 8.5	27.5 ± 10.1

Quantitative variables are presented as the mean ± standard deviation, and non-parametric variables in the histogram are expressed as the median [interquartile range]. Abbreviations: BMI, body mass index; MNA-SF, Mini Nutritional Assessment Short Form; SMI, skeletal muscle mass index; MASA, Mann Assessment of Swallowing Ability; FIM, Functional Independence Measure.

**Table 2:** Amount of nutrition and intervention during the study.

Variable	All (*n* = 95)	Female (*n* = 76)	Male (*n* = 19)
The mean energy intake, kcal/kg/day	29.1 ± 4.9	28.9 ± 5.1	29.7 ± 4.0
Patients consuming ≥30 kcal/ kg/day, n (%)	39 (41.0)	30 (39.5)	9 (47.4)
The mean protein intake, g/kg/day	1.09 ± 0.18	1.09 ± 0.18	1.12 ± 0.15
Patients consuming ≥1.2 g/kg/day, n (%)	26 (27.3)	22 (28.9)	4 (21.1)
Length of days for intervention	60 [53–60]	60 [53–60]	60 [47–60]
Mean intervention time per day, min	129.6 ± 18.1	130.2 ± 18.9	126.8 ± 14.3
Physical therapy	93.0 ± 17.6	93.1 ± 18.2	92.7 ± 15.7
Occupational therapy	36.5 ± 13.3	37.2 ± 12.6	34.1 ± 15.8

Quantitative variables are presented as the mean ± standard deviation, and non-parametric variables in the histogram are expressed as the median [interquartile range]. Energy and protein intake are presented as mean intake per ideal body weight [kg] per day.

**Table 3:** Swallowing and physical outcomes before and after the intervention.

Variable		All		*p* Value		Female			Male	
	Before	After	Mean Difference between Pairs [95% CI]		Before	After	Mean Difference between Pairs [95%CI]	Before	After	Mean Difference between Pairs [95% CI]
All (*n* = 95)					(*n* = 76)			(*n* = 19)		
MASA, score	191.8 ± 5.7	193.0 ± 5.4	1.12 [0.53–1.70]	<0.001	191.8 ± 6.19	193.1 ± 5.8	1.27 [0.64–1.92]	191.8 ± 3.4	192.3 ± 3.6	0.47 [−1.00–1.94]
FIM total, score	65.9 ± 14.5	96.8 ± 20.1	30.9 [28.1–33.7]	<0.001	64.9 ± 13.9	97.0 ± 19.4	30.5 [27.4–33.7]	70.0 ± 16.3	95.9 ± 23.5	32.3 [25.6–39.0]
Patients with decreased tongue strength at baseline (*n* = 30)					(*n* = 27)			(*n* = 3)		
MASA, score	186.8 ± 5.7	189.7 ± 6.6	3.00 [1.71–4.23]	<0.001	186.6 ± 5.9	189.5 ± 6.9	2.9 [1.5–4.3]	186.8 ± 5.7	189.7 ± 6.6	3.6 [0.8–6.5]
FIM total, score	62.9 ± 15.0	93.8 ± 22.1	30.9 [25.9–35.9]	<0.001	67.3 ± 14.2	98.2 ± 19.2	31.3 [26.7–36.0]	62.9 ± 15.0	93.8 ± 22.1	33.3 [0.5–66.1]

Abbreviations: MASA, Mann Assessment of Swallowing Ability; FIM, Functional Independence Measure; CI, confidence interval.

**Table 4:** Impact on tongue strength after intervention.

	Impact on Changes in Tongue Strength *		High energy Intake on Tongue Strength ^†^		High Protein Intake on Tongue Strength ^†^		Physical Therapy Time on Tongue Strength ^†^	
Variable	Coefficient ^‡^	*p* Value	Coefficient ^‡^	*p* Value	Coefficient ^‡^	*p* Value	Coefficient ^‡^	*p* Value
Age	−0.071 (−0.231, 0.089)	0.379						
Male	2.021 (−0.559, 4.600)	0.123	−0.080 (−3.075, 2.916)	0.958	0.407 (−2.579, 3.393)	0.787	0.633 (−2.517, 3.783)	0.691
BMI	0.180 (−0.178, 0.538)	0.321						
MNA-SF	0.073 (−0.416, 0.562)	0.767						
Hand-grip strength	0.328 (0.113, 0.544)	0.003	0.268 (0.019, 0.516)	0.035	0.259 (0.009, 0.510)	0.043	0.266 (0.003, 0.528)	0.048
Male	0.320 (−0.190, 0.829)	0.202						
Female	0.317 (0.013, 0.620)	0.041						
SMI	1.318 (0.044, 2.592)	0.043	0.648 (−0.938, 2.234)	0.419	0.625 (−0.971, 2.220)	0.439	0.223 (−1.431, 1.878)	0.789
Male	0.769 (−4.611, 6.149)	0.766						
Female	1.264 (−0.332, 2.861)	0.119						
MASA at admission	0.233 (0.026, 0.440)	0.028	0.237 (0.041, 0.432)	0.018	0.256 (0.059, 0.453)	0.012	0.225 (0.020, 0.429)	0.031
FIM score at admission	0.019 (−0.055, 0.092)	0.505						
Motor component	0.031 (−0.061, 0.122)	0.930						
Cognitive component	−0.009 (−0.210, 0.192)	0.616						
Length of days from onset to hospitalization	−0.027 (−0.092, 0.037)	0.404						
Consuming ≥30 kcal/kg/day	2.407 (0.356, 4.457)	0.022	2.553 (0.598, 4.508)	0.011				
Consuming ≥1.2 g/kg/day	2.073 (−0.219, 4.364)	0.076			2.613 (0.430, 4.796)	0.020		
Mean intervention time per day, min	0.716 (−0.446, 1.878)	0.224						
Physical therapy intervention time per day	0.939 (−0.238, 2.116)	0.117					0.017 (−0.044, 0.078)	0.575
Occupational therapy intervention time per day	−0.341 (−1.911, 1.229)	0.667						

* Univariate analysis. † Multivariate analysis. ‡ Coefficients and the lower and upper 95% confidence intervals are computed in each regression model against tongue strength after the 2-month intervention adjusted according to tongue strength before intervention.
